# Representation of Organ Transplantation in Cinema and Television

**Published:** 2011-05-01

**Authors:** G. G. Kalra, D. Bhugra

**Affiliations:** 1 *Assistant Professor, Department of Psychiatry, LTMMC and LTMGH, Sion, Mumbai, India*; 2 *Professor of Mental Health and Cultural Diversity, Health Service & Population Research Department, Institute of Psychiatry, King’s College London, UK*

**Keywords:** Attitudes, Films, Media, Organ transplantation, Television

## Abstract

Media whether print or visual such as films and television remains an important source of information and education for the general population even if it is not meant to be such. Films in particular have significant impact on the individual psyche. Films are meant for entertainment but it is inevitable that they will reflect the attitudes of society and in turn will influence the way societies and their members perceive conditions. In this paper we describe the use of films in making audiences aware of issues related to organ-donation. We review how films have dealt with the issue of organ transplantation over the years and suggest that a positive portrayal of organ transplantation in films and other media channels will allay negative attitudes in people and may act as catalysts of behavior change. This can motivate more people to donate organs posthumously. The portrayals of the act itself, its sequelae for the recipient and the donor’s families will be discussed.

## INTRODUCTION

Cinema intentionally or otherwise can act as a useful source for educating people and influencing their perception of specific or general issues related to any topic. Of course both media and cinema can do harm as well by providing stereotypes and distortions in the cause of telling a good story. Organ transplantation is one of the most complex medical achievements till date and has received much media attention since the father of transplantation, Thomas E. Starzl performed the first human liver transplant in 1963 and the first successful liver transplant in 1967. Cinema portrayals of organ transplantation are likely to affect public views and at the same time, reflect the public views at that point in time. Apart from the print media, films and recently television programs in many different countries have dealt with organ transplantation in their storylines in various ways, sometimes positive and negative at other times. It is likely that the portrayals of this sensitive issue in the films may play an active role in maintaining positive and negative beliefs about this complicated surgical procedure in public minds. 

**ORGAN TRANSPLANTATION: A BACKGROUND**

The field of organ transplantation is by far the only procedure that offers a hope of better quality of life for people with end-stage organ failures. The field has undergone tremendous progress not only in the organs that can be transplanted but also in the techniques used and medical specialties involved. A long waiting period for an appropriate donor may lead to distress [1] as much as complications resulting from various medical procedures and drug treatments [2]. The unpredictable outcome of the transplantation procedures creates a fertile emotional soil for psychiatric complications [3]. On the other hand, previously psychiatrically ill patients may also require transplantation. And, hence the need of the mental health professional arises in this medical field giving rise to Transplantation-Psychiatric Consultation (TPC). Psychiatrists working with such individuals may have to deal with issues ranging from something as minor as anxiety about the surgical procedure to the fear of death and organ rejection apart from other psychiatric disorders that may arise. 

There is a vital need for donated organs and it is worth noting that although this need has increased many folds, the supply is yet to be met. A look at the organ donor waiting-list data from various countries highlights the plight of the condition. For example the number of White individuals waiting for organs, increased 146% from 1993 to 2002, while for the same period, there was a 260% increase for Hispanic Americans [4]. This scenario is no different in other parts of the world. But the supply of organs is still the same and cannot meet the ever-increasing demand. 

The reasons for such a discrepancy between demand and supply are many. Evidence suggests that people may express reluctance to agree to the donation of a family member’s organs due to lack of awareness regarding the deceased’s wishes [5], which may indicate that there is lesser of family discussions on organ donation. Many family members tend to have mixed feelings about opening up of their relative’s body, and worry that it is disrespectful and may disturb the peace of the dead [6]. Also posthumously, the family members may be grieving, which does not seem to be the appropriate moment to talk to them about organ donation. It is also inevitable that attitudes to organ transplantation will be influenced by cultural factors and values [6]. Based on cultural beliefs that are often intertwined with religious traditions, people may conduct particular burial and funeral rites that do not allow for organ donation. The Japanese, for instance, traditionally believe that the dead body must remain whole because the soul would become unhappy in the next world if his organs are removed in this one, given that there is a fragment of the deceased’s mind and spirit in every part of the dead body [7]. This is also the case with the Chinese [8] and Gypsies or the Romans who too are averse to organ transplantation because they have faith that the human being must be left intact upon death for a good afterlife [9]. Contrary to this, Christians simply treat organ transplantation as an act of love [10]. 

People may view body organs as an essential part of their bodies and identities, their integrity. Donating organs thus would mean doing away with this bodily integrity, removing away the pieces of who you are (in case of the donor) and making the recipient lesser of himself or herself. Different people may be motivated by different motives to donate organs. More interesting is to understand these motives, both conscious and unconscious, in living donors. The fact that a healthy volunteer exposes himself or herself to the risk of surgery solely for the benefit of another individual makes living organ donation unique. Some donors may view the idea of living through another person even after death by donating their organs. Some family members may donate organs of their deceased kin to ensure that the organs can benefit others instead of merely decomposing and becoming biological waste [6]. The decision to donate might be motivated by attempts to make reparation for wrongs committed in the past or to secure a commitment from the recipient [11]. Others may sell their organs for money to overcome their financial problems. This has given rise to the illegal organ trade throughout the world, exposing the poor and vulnerable individuals to donate for monetary gains. The “availability” of organs refers to what is defined as legally permissible and morally acceptable, which in turn reflects the cultural givens of that particular cultural group [6]. 

**CINEMA AND ORGAN TRANSPLANTATION**

Over the years, various films have dealt with the subject of organ transplantation, highlighting different aspects. Some of these sub-genres have been made as comedies while others have shown the tragic side of it; still others have taken the horror route to deal with the subject. Films can present truths or can promote myths about donation. Films based on organ transplantation can be traced on a timeline into three eras (Table 1): those that came prior to the 1990s (Era I), those that came in the decade from 1990–2000 (Era II), and the films post-2000 (Era III). The films screened by the authors are a personal choice and may not include all films based on this issue. The portrayal of transplantation and related psychiatric aspects in these films seem to have evolved alongside that of social attitudes to psychiatry and more so to the level of involvement of psychiatry as a discipline with the field of transplantation. One does not see any psychosocial aspects of organ transplantation being covered in the first era films, and a slow appearance of such coverage in the post-2000 era cinema. 

**Table 1:** Timeline of films on organ transplantation

**Era I (Pre-1990s)**			**Era II (1990–2000)**			**Era III (Post-2000)**		
**Year**	**Film**	**Organ**	**Year**	**Film**	**Organ**	**Year**	**Film**	**Organ**
1940	Black Friday (H)	Brain	1992	Dr. Giggles (H)	Heart	2003	21 Grams (T)	Heart
1960	Eyes Without a Face (H, T)	Face	1995	Donor Unknown (T)	Heart	2007	Recycled Parts (T)	Multiple organs
1962	The Awful Dr. Orloff (H,T)	Skin	1997	Face-off (A)	Face	2008	The Harvest Project (T)	Multiple organs
1962	The Brain That Wouldn’t Die (H,T)	Brain	1997	Lifebreath (T)	Lung	2008	Seven Pounds (D)	Multiple organs
1963	Doctor of Doom (H,T)	Brain	1998	Nicholas’ Gift (D)	Multiple organs	2008	The Eye (T)	Cornea
1964	Monstrosity (or The Atomic Brain) (H,T)	Brain, Xeno-transplantation	1999	Heart (D)	Heart	2009	My Sister’s Keeper (D)	Bone marrow among others
1968	Night of the Bloody Apes (H,T)	Heart, Xeno-transplantation	2000	Return to Me (D)	Heart	2009	Tell Tale (T)	Heart
1971	The Incredible Two-Headed Transplant (H,T)	Head						
1971	Brain of Blood (H,T)	Brain						
1974	Young Frankenstein (H)	Brain						
1976	Mansion of the Doomed (H)	Eyes						
1983	The Man with Two Brains (C)	Brain						
1988	Faceless (H,T)	Face						

H : Horror;

T : Thriller;

D : Drama;

C : Comedy;

A : Action

A common theme that seems to be running in the pre-1990 era films on organ transplantation is the negative portrayal of the transplant surgeons who are motivated by tragedies in their personal lives for instance, accidents, in which their wives have either died or been physically disabled as in *The Awful Dr. Orloff* (1962) and *The Brain That Wouldn’t Die* (1962). This sets the plot for the surgeons to start doing desperate and unhindered transplants, turning into abductors, murderers and lunatics in the whole process. However, such depictions are often violent and gory. Most of these films deal with the impossible-looking brain and head transplantations and usually follow a horror or a thriller route, often showing psychosis to be a common outcome of the transplant procedures, wherein the recipient turns into a monster, thus conveying to the viewer that transplants lead to negative perhaps psychotic and violent outcomes in the recipient (e.g., *Night of the Bloody Apes* (1968)). It is true that adjustment after transplant surgery can be a stressful experience [12] but psychotic outcome is not necessarily a ubiquitous phenomenon, something which has been projected in these films. *Black Friday* (1940) and* The Brain That Wouldn’t Die* (1962) are probably the earliest films based on transplantation featuring brain transplant. The former film shows multiple personality disorder and psychosis developing in the patient post-transplant. Out of the films the authors screened from Era I, only one film (comedy), *The Man with Two Brains* (1983), dealt with brain transplantation in a somewhat positive light and the rest all films showed transplant in a negative way (Table 1).

Films from the 1990–2000 decade (Era II) were influenced by heart transplants and the emotions that arise thereafter. Most of the heart transplant portrayals were again negative, except for films like *Return to Me* (2000) which show how one can get deeply involved with this procedure; heart being seen as the symbolic seat of love and loyalty may be one of the reasons for such deep involvement of those affected [13, 14]. The film subtly raises the issue of writing anonymous gratitude letters to the donors’ family by the recipients and how they can get involved in each other’s lives. In fact, families involved in cadaveric donation receive little follow-up from hospitals after their ordeal. No letters of thanks are sent to the bereaved from the hospital authorities or those who receive transplants. This has given rise to some donor next of kin considering themselves as being part of an invisible and unrecognized minority [6]. *Face Off* (1997) portrayed the negative impact that face transplantation can have in the lives of those involved by misusing one’s identity as face is an intrinsic and a vital part of an individual’s identity. The psychological impact of face transplantation needs to be looked into seriously since it is now soon becoming a reality. 

Post-2000 (Era III), as the possibility of wider organs for transplantation grew, the sophistication of the transplant procedures also increased, and psychiatry became an important part of the multi-disciplinary transplant team. Work by various researchers demonstrated that individuals seen in a transplant setting differ from those seen in general hospitals and are more likely to have psychiatric issues [1, 15]. Trzepacz, *et al*, (1989) [16] found that 20% of liver transplant candidates had concomitant adjustment disorders and 4.5% had major depression; 9% met the criteria for alcohol abuse or dependence. Similarly, major depression has been described in 5% to 22% [17, 18] of patients undergoing dialysis. With increasing recognition of these mental-health issues, the role of psychiatrist for pre-transplant assessment of both donors and recipients became important and simultaneously a lot of experimentation was seen with the portrayal of transplantation in films. Areas like family dynamics, consent issues and organ black market came to be explored in certain films in this era. 

*Seven Pounds* (2008) is a recent film which deals with multiple organ donations; the lead character donating his eyes, heart, bone marrow, kidney, and a part of his lung and liver, to six different people, in a bid for redemption for the deaths of seven people that he causes accidentally. The lead character seems to be suffering from depression and survivor guilt and commits suicide in the end, with the final donation of his heart to his girlfriend. However, what would have been interesting is the aftermath of the story. The film ends with the girlfriend realizing the altruistic donation of her boyfriend to her and other people in need, which is one of the many motivations for donation [19]. This is a significant observation in that post-transplant feelings need to be explored at length. For instance, kidney recipients report that they may not pursue living donation because they feel guilty and indebted to the donor and do not want to harm or inconvenience the donor [20]. 

*Seven Pounds* projects the concept of organ donation in both good and bad lights. It gives a positive view of the donor as saving the lives of those in need, but at the same time it does this at the cost of the donor’s life who kills himself, which could promote the myth that it is acceptable to take one’s own life to save that of the other. 

A decade earlier to *Seven Pounds*, another film from Era II, *Nicholas’ Gift* (1998) portrayed multiple organ donation in a positive light giving it a social meaning of a “gift” [21]. Gift exchange is a theory governed by the principles of giving, receiving and reciprocating and has many similarities with the process of organ transplantation [22], giving a logical explanation to the experiences of donors and recipients. Based on true story, the film deals with the issue of organ transplantation following death delicately, breaking the taboo and appears to be a watershed film between the older sub-genre and the newer ones. 

Another interesting film from Era III, *My Sister’s Keeper* (2009) specifically addresses the psychiatric aspects and family dynamics involved in organ donation; the younger and legally minor daughter being the donor to the elder and terminally ill sister. The film shows the emotional responses like ambivalence, passivity, and denial of the severity of her illness in the family recipient, and that of ambivalence and concerns of being coerced into donation, in the family donor, that is the younger daughter [21]. The film raises the ethical question of consent and how far one can go to save the life of a terminally ill individual, while risking that of the other. As per the WHO guiding principles on organ transplantation (2010), while the permission of parent(s) or the legal guardian for organ removal is usually sufficient, they may have a conflict of interest if they are responsible for the welfare of the intended recipient, which is what is portrayed in *My Sister’s Keeper*. In such cases, review and approval by an independent body, such as a court or other competent authority, should be required. In any event, a minor’s objection to making a donation should prevail over the permission provided by any other party [23]. 

Although rare, films have also dealt with other organ transplants like skin, face and corneas (Table 1). Two films from Era I and III, *Mansion of the Doomed* (1976) and *The Eye* (2008) respectively deal with corneal transplantation and have horror running through their plot. Since research has pointed out a significant relationship between stigma and perceived dangerousness [24], the plots of these and such other films are likely to encourage an anti-organ-donation attitude and will give rise to stigmatizing views in the public. 

**TELEVISION AND ORGAN TRANSPLANTATION**

A television programme is primarily considered a medium of journalism and only secondarily as a public service [25]. Television seems to affect a larger audience and through two important channels—long running series and advertisements. 

Long running series may affect individuals and their attitudes at a deeper level, since they may start relating to the characters in these series simply due to the duration that these series remain on air. *Three Rivers* (2009–2010) is one such series based on lives and situations of organ donors, recipients and the treating teams. The authors recommend that programs like these may be used in sending positive messages to the audience regarding sensitive issues like transplantation. *De Grote Donorshow* (2007) was a hoax reality TV program broadcast in the Netherlands which despite receiving heavy criticism led to a substantial increase in people consenting for organ donation [26]. 

Similarly suitable television advertisements may be associated with pro-donation attitudes, but an argument put forward is that pro-donation individuals may be more likely to either view or remember the television advertisements portraying organ donation. It is advisable to place such programs and advertisements strategically in prime-time schedules, so as to have a maximum impact. Roping in celebrities as brand ambassadors for such advertisements would help the policy makers in their endeavor to pass on the message. 

**ROLE OF MEDIA IN CHANGING ATTITUDES**

Media and specifically films are best suited to introduce a novel idea to a population and, create a social context in which this idea becomes the focus of community discussion [27-29]. In case of organ transplantation, studies have reported that education is the best way to reach living donors and dispel fears [19], and media is an important educational tool that has a powerful impact on public attitudes [30, 31]. Films definitely have power to influence the public perception about various social issues. Individuals watching films or television are not passive agents, but active interpreters of the covert and overt messages in the storylines, who later on enact these learned messages in their lives. Thus education efforts may also be mediated by characteristics of the program viewers [32]. 

The media may depict a world in which unhealthy behaviors such as physical aggression, unprotected sex, smoking and drinking are glamorous and risk-free [33]. Various studies document that exposure to media may result in increased violent and aggressive behavior [34] and disordered eating behaviors [35, 36], while it may influence tobacco cessation in adults [37]. Adolescents who perceive greater support from the media for teen sexual behavior, report greater intentions to engage in more sexual activity [38] and this media may include music, movies, television, and magazines [39]. 

Alvaro, *et al*, [40, 41] noted differences between respondents in their study who were exposed to a mass media campaign on living organ donation and those in the same community who were not exposed to the campaign, with the exposed respondents reporting more positive living organ donation behavioral intentions than the non-exposed respondents. The media campaign included television and radio advertisements. The study by Alvaro, *et al*, [40] was done on Hispanic population, since Hispanic Americans are 60% less likely to donate their organs [42, 43]. But this same scenario can be extrapolated to the general population and it can be said that people are not very keen on donating their organs. 

Alvaro, *et al*, [44] pointed that the fear of donation process and lack of knowledge or information were the main barriers to living organ donation. Films like *The Eye* (2008) may negatively impact the public and instill fear about the transplantation process, decreasing the recipient’s pursuit of organ donation. Table 2 enumerates some desirable qualities that can be incorporated in films portraying issues surrounding organ transplantation.

**Table 2:** Desirable qualities in films based on Organ Transplantation

Should normalize the transplant experience
Ethical issues
Non-violent, non-suicidal
Motivate people to become organ donors
Instill positive attitudes in the viewers about the issue
Inform and educate viewers about the process
Entertaining to watch

However, one has to be aware that even though cinema is a strong tool for sending a message across, it may not be able to break certain stereotypes in the society, which may be relatively immutable to the effects of education [45, 46]. 

## CONCLUSION

Films and other television programs have evolved in their coverage of organ transplantation both in qualitative and quantitative terms. One can see a definite shift in the variety of films that portray this issue, alongside the attitudes to psychiatry and psychosocial attitudes to organ transplantation. Cinema is an important educational tool and it is important that cinema takes up the issue of portraying organ transplantation in a responsible way and positive light so that people understand the necessity of donating organs. Film influences should be considered in research and interventions with organ donors and recipients to reduce the gap between them. Donor willingness and ultimately actual organ donation should be the desired intervention outcomes of any media programs or films. Harnessing the power of media and films in particular appropriately is the need of the hour. 
